# Omega 3 polyunsaturated fatty acid improves spatial learning and hippocampal Peroxisome Proliferator Activated Receptors (PPARα and PPARγ) gene expression in rats

**DOI:** 10.1186/1471-2202-13-109

**Published:** 2012-09-18

**Authors:** Toktam Hajjar, Goh Y Meng, Mohamed A Rajion, Sharmili Vidyadaran, Fauziah Othman, Abdoreza S Farjam, Tan A Li, Mahdi Ebrahimi

**Affiliations:** 1 Department of Veterinary Preclinical Sciences, Universiti Putra Malaysia, 43400 UPM, Serdang, Selangor, Malaysia; 2 Institute of Tropical Agriculture, Universiti Putra Malaysia, 43400 UPM, Serdang, Selangor, Malaysia; 3 Department of Pathology, Faculty of Medicine and Health Sciences, Universiti Putra Malaysia, Serdang, Selangor, Malaysia; 4 Department of Human Anatomy, Faculty of Medicine and Health Sciences, Universiti Putra Malaysia, Serdang, Selangor, Malaysia

**Keywords:** PUFA, n-6: n-3 PUFA ratio, Spatial learning, PPAR, Cognitive function

## Abstract

**Background:**

This study examined the effects of dietary polyunsaturated fatty acids (PUFA) as different n-6: n-3 ratios on spatial learning and gene expression of peroxisome- proliferator-activated receptors (PPARs) in the hippocampus of rats. Thirty male *Sprague–Dawley* rats were randomly allotted into 3 groups of ten animals each and received experimental diets with different n-6: n-3 PUFA ratios of either 65:1, 22:1 or 4.5:1. After 10 weeks, the spatial memory of the animals was assessed using the Morris Water Maze test. The expression of PPARα and PPARγ genes were determined using real-time PCR.

**Results:**

Decreasing dietary n-6: n-3 PUFA ratios improved the cognitive performance of animals in the Morris water maze test along with the upregulation of PPARα and PPARγ gene expression. The animals with the lowest dietary n-6: n-3 PUFA ratio presented the highest spatial learning improvement and PPAR gene expression.

**Conclusion:**

It can be concluded that modulation of n-6: n-3 PUFA ratios in the diet may lead to increased hippocampal PPAR gene expression and consequently improved spatial learning and memory in rats.

## Background

The n-3 polyunsaturated fatty acids (PUFA) play an important role in cellular functioning and normal brain cognitive function development including learning and memory [1,2]. The studies have shown that low dietary n-3 PUFA and low plasma docosahexaenoic acid (DHA; 22:6 n-3) concentrations decreased n-3 PUFA in the brain and in turn resulted in behavioral defects [3,4]. It is known that high dietary saturated fat lead to hypercholesterolemia, and high blood pressure, causing atherosclerosis [5]. Hypertension and atherosclerosis cause dysfunction in the blood brain barrier function, and ischemia in the brain which may lead to the cognitive impairment [6-8]. On the contrary, recent studies have shown that PUFA improve spatial memory [5,9]. The PUFA supplementation contributes to modulation of membrane composition, fluidity and selective permeability, cellular signaling and regulation of gene expression [10].

The current diets are generally deficient in n-3 fatty acids and abundant in n-6 [11]. The n-3 fatty acids increase membrane fluidity by replacing n-6 fatty acids and cholesterol from the membrane [12] maintaining an optimal membrane fluidity which is obligatory for neurotransmitter binding and signaling within the cell [13]. The n-3 PUFA incorporated into the neuron membrane also increase synaptic protein expression, strengthening the hippocampal synaptic plasticity [3]. This is modulated by transcription factors such as peroxisome proliferator-activated receptors (PPARs) [14].

The PPARs play a critical physiological role as lipid sensors and regulators of lipid metabolism [15]. The PPARα and PPARγ are the key messengers responsible for the translation of nutritional stimuli into changes for the expression of genes, particularly genes involved in lipid metabolism [16]. Fatty acids can activate and induce the enzymes of the peroxisomal β-oxidation pathway at the transcriptional level by the mediation of ligand-activated transcription factors PPARs [17,18]. The PPARα plays an important role in the regulation of acetylcholine biosynthesis that contributes to cognitive function [14,19], and PPARγ has a prominent role in the regulation of central nervous system (CNS) inflammation and neuroprotection [20,21] leading to improvement in cognitive performance [20,22]. These findings indicated that cognitive performance can be enhanced through PPAR nuclear receptors [23]. However, the modulation effects of different dietary n6: n-3 ratio on PPAR genes at the hippocampal level is still not well understood. Therefore, the present study aimed to examine the hypothesis that a reduction in dietary n-6: n-3 PUFA ratios may alter the hippocampal PPAR gene expression and consequently spatial memory.

## Results

### Behavioral test

#### Spatial acquisition

**Figure 1:**
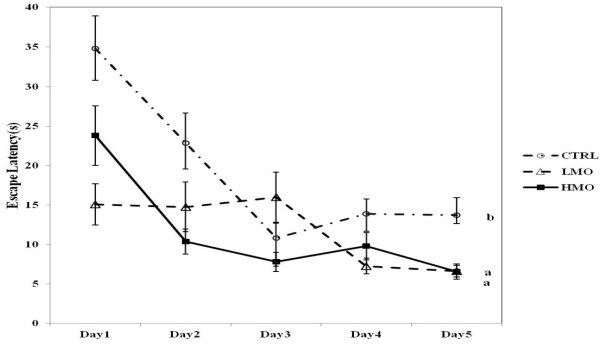
**Escape latency over the training period (n = 10).**^a,b^ Lines with different alphabetic notation differ significantly at *P* < 0.0.5. CTRL, LMO and HMO refer to groups of rats fed with control, low menhaden oil, and high menhaden oil, respectively.

On the first trial of acquisition, no difference was found in escape latency among the three groups. The relative escape latency of all groups gradually declined over the training period (*P* < 0.05) (Figure 1). On the first day of the second trial, the LMO and HMO rats had a shorter relative escape latency compared with CTRL (*P* < 0.05). On day 5, the HMO rats spent the shortest time to find the platform (*P* < 0.05).

#### Probe trial

**Figure 2:**
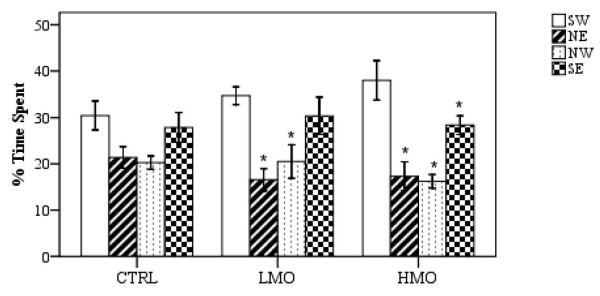
**Relative time spent in each quadrant in the probe test (n = 10).** Significant differences from the mean values of the target quadrant (SW) in each group were indcated by * (*P* <0.05). Error bar shows ±1SE, CTRL, LMO and HMO refer to group of rats fed with control, low menhaden oil (high n-6: n-3), and high menhaden oil (low n-6: n-3), respectively. SW: south-west, NE: north-east, NW: north-west, SE: south east.

The HMO rats swam for a higher percentage of time in the target quadrant (SW) compared with the other quadrants (*P* < 0.05) (Figure 2). Conversely, the CTRL rats relied on random probability to locate the platform and spent a similar percentage of time in all quadrants (*P* > 0.05).

### Gene expression

**Figure 3:**
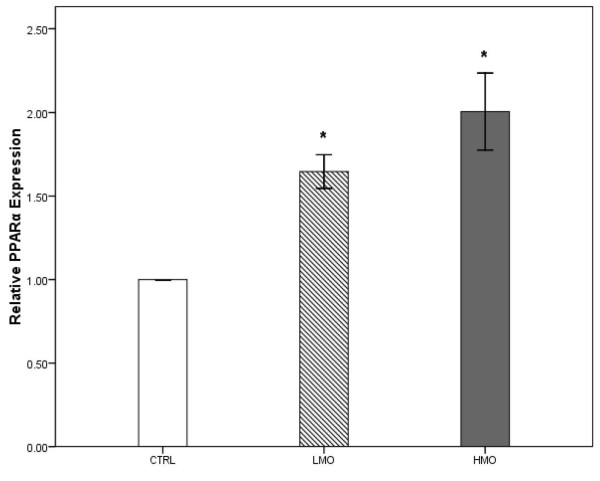
**Expression of PPARα in the brain of treatment groups compared to CTRL group.** Triplicates of real-time PCR were performed for each sample. Values were normalized with a housekeeping gene, β-actin. Then, treated samples were expressed relative to gene expression of CTRL group. Statistical analysis was perfomed using a Student’s t test. Values are means ± 1 standard error bar. Values indicated by * show significant differences compared with the CTRL group (*P*<0.05). CTRL: control, LMO: low menhaden oil, HMO: high menhaden oil.

**Figure 4:**
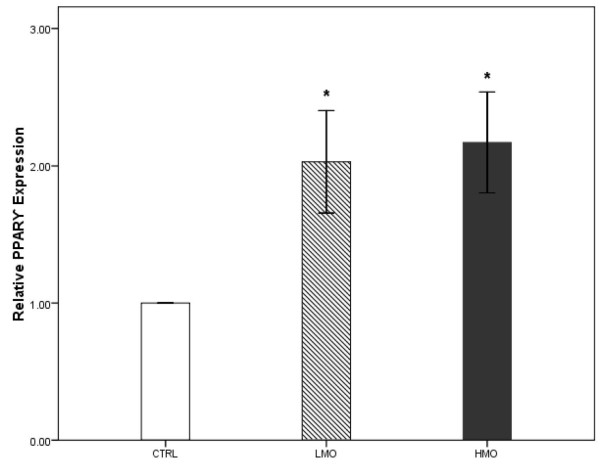
**Expression of PPARγin the brain of treatment groups compared to CTRL group.** Triplicates of real-time PCR were performed for each sample. Values were normalized with a housekeeping gene, β-actin. Then, treated samples were expressed relative to gene expression of CTRL group. Statistical analysis was performed using a Student’s t test. Values are means ± 1 standard error bar. Values indicated by * show significant differences compared with the CTRL group (*P* <0.05). CTRL: control, LMO: low menhaden, HMO: high menhaden oil.

The effects of different levels of menhaden fish oil and soybean oil supplementation on PPAR gene expression are shown in Figure 3 and 4. The PPARα (Figure 3) and PPARγ (Figure 4) genes showed a higher level of expression in the LMO and HMO groups compared with the CTRL group (*P <* 0.05). In other words, the reduction of dietary n-6: n-3 PUFA ratios upregulated the PPAR gene expression in the hippocampus.

## Discussion

The aim of the present study was to clarify whether the n-3 fatty acids could affect the expression of mRNA encoding for PPARs in the memory-related brain region, the hippocampus. Previous studies have reported that dietary PUFA were able to increase PPARα and PPARγ gene expression in different tissues. For instance, the expression of PPARα genes in human macrophages tends to increase following incubation with n-3 fatty acids [24]. The studies have also reported that the increasing dietary PUFA will upregulate PPARα and PPARγ expression after lipopolysaccharide (LPS)-induced PPARs downregulation in the spleen, liver and bursa of chickens [25].

In this study, the results demonstrated that diets with low n-6: n-3 PUFA ratios improved spatial memory and upregulated the hippocampal PPARα and PPARγ gene expression. This is evident in the performance of the unsupplemented CTRL group in the probe trial. The CTRL group had difficulties recalling the location of the escape platform from the most recent training session. As a result they depended on random chance to locate the platform and spent a similar percentage of time in all quadrants. The probe trial is typically used to assess reference memory in test subjects, and administered immediately after a training or acquisition session [26]. Current results also indicated differences between the expression of PPARα and PPARγ genes in the rats supplemented with two different n-6: n-3 PUFA ratios. In fact, the level of expression of these genes was increased by the reduction of n-6: n-3 fatty acid ratios. The PPARα and PPARγ are expressed widely in the CNS, especially in the brain areas involved in learning and memory, such as the hippocampus [27]. The anti-inflammatory and anti-apoptotic role of PPARs could play important functions in the improvement of the mental ability of the brain [14]. Other possible mechanisms underlying the links between these changes in PPARs expression and the learning process may be related to their transcriptional role in synaptic plasticity and long term potentiation (LTP). The involvement of these transcription factors in protein synthesis are relevant to the synaptic mechanisms that mediate the formation of long term memory. Transcription is an essential step for long term synaptic plasticity and transcription factors play an important role in LTP through molecular alterations which facilitate long term synaptic plasticity and memory formation [28,29]. Indeed, participation of the transcription factor PPARs in the consolidation of memory in the rat’s hippocampus results in the improved performance in the spatial memory task. The PPARs have specific functions in regulating the expression of genes involved in neurotransmission, and therefore play important roles in complex processes including learning and memory formation [14]. They played an essential role in regulating gene encoding for neurotransmitter receptors, synaptic protein and enzymes involved in neurotransmitter metabolism or release [14]. The n-3 PUFA stimulate PPAR target gene expression such as lipoprotein lipase (LPL), fatty acid transport protein (FTTP) and acyl-CoA synthase (ACS), thus enhancing the effects of n-3 PUFA on brain function [29].

In summary, the study showed that there is a relationship between gene expression of PPARs and feeding with n-3 fatty acids. The increase in n-3 PUFA of the brain may lead to increased PPAR gene expression. It also suggests that PPARα and PPARγ as transcription factors may affect the expression of synaptic proteins leading to increased synaptic plasticity and improved spatial memory in animals supplemented with higher n-3 PUFA, where the ratio of n-6: n-3 of 4.5:1 was more effective than the ratio of 22:1.

## Conclusions

In this study, it was shown that the n-3 PUFA supplementation resulted in differences in PPARα and PPARγ expressions in the brain. In conclusion, PPARα and PPARγ genes displayed a tendency to be expressed at a higher level in rats fed diets with a lower n-6: n-3 PUFA ratio and may be able to improve spatial learning and memory. Further studies should be done using relatively pure n-6 and n-3 fatty acids to confirm these findings, and confirm that the lower n-6: n-3 ratios can improve learning and memory and increase the expression of PPARs in the hippocampus. Further investigations should be carried out to examine the effects of n-6: n-3 ratios on the gene expression in other brain regions such as the prefrontal cortex, amygdala and others.

## Methods

### Animals and diets

Thirty individually housed, three-week old male *Sprague–Dawley* rats were allotted randomly into three groups of ten animals each namely the control (CTRL), low menhaden oil (LMO), and high menhaden oil (HMO) groups. They were maintained under a light/dark cycle 12/12 h at constant temperature (25 ±2°C) and humidity (50–60%). The experiment was approved by the Institutional Animal Care and Use Committee (IACUC) of the Faculty of Veterinary Medicine, Universiti Putra Malaysia. After two weeks of adaptation, the rats were fed for 12 weeks as follows: CTRL, standard pellet enriched with 7% (w/w) of butter; LMO, standard pellet enriched with 1% (w/w) fish oil + 6% (w/w) soybean oil; HMO, standard pellet enriched with 3.5% (w/w) fish oil + 3.5% (w/w) soybean oil (Table 1). All diets were prepared fresh and fed to the animals once daily.

**Table 1:** Fatty acid profile of experimental diets (% of total identified fatty acids)

	**Experimental diets**		
**Fatty acid**	**CTRL**	**LMO**	**HMO**
Caprylic Acid (8:0)	0.70	0.17	0.13
Capric Acid (10:0)	0.87	0.13	0.09
Lauric Acid (12:0)	6.05	0.06	0.05
Myristic Acid (14:0)	4.66	1.26	2.82
Palmitic Acid (16:0)	28.11	15.16	16.47
Palmitoleic Acid (16:1)	0.84	1.53	3.93
Stearic Acid (18:0)	6.77	4.18	4.84
Oleic Acid (18:1n-9)	29.59	20.35	26.85
Linoleic Acid (18:2n-6)	17.78	40.37	31.94
Linolenic Acid (18:3n-3)	0.28	0.37	0.27
Arachidic Acid (20:0)	1.51	2.09	2.39
Arachidonic Acid (20:4n-6)	0.15	0.13	0.29
Eicosapentaenoic acid (20:5n-3)	nd	0.92	3.40
Docosapentaenoic acid (22:5n-3)	nd	0.20	1.13
Docosahexaenoic acid (22:6n-3)	nd	0.45	2.46
Total SFA	50.19	24.43	27.98
Total UFA	49.81	75.57	72.02
Total MUFA	31.54	33.03	32.29
Total n-3 PUFA	0.28	1.94	7.26
Total n-6 PUFA	17.99	40.59	32.47
n-6: n-3	64.25	20.92	4.47
UFA:SFA	0.99	3.09	2.57
PUFA:SFA	0.36	1.74	1.42

CTRL: control, LMO: low menhaden oil, HMO: high menhaden.

nd: not detected.

Total SFA: sum of 8:0, 10:0, 12:0, 14:0, 16:0, 18:0 and 20:0.

Total UFA: sum of 16:1, 18:1n-9, 18:2 n-6, 20:4n-6, 18:3n-3, 20:5n-3, 22:5n-3 and 22:6n-3.

Total MUFA: sum of 16:1 and 18:1n-9.

Total n-3PUFA: sum of 18:3n-3, 20:5n-3, 22:5n-3 and 22:6n-3.

Total n-6PUFA: sum of 18:2n-6, 20:4n-6.

n-6: n-3 :Total n-6 PUFA: (sum of 18:2n-6, 20:4n-6)/ Total n-3 PUFA: (sum of 18:3n-3, 20:5n-3, 22:5n-3 and 22:6n-3).

### Morris water maze

At the end of the 10^th^ week, all rats were assessed for their spatial memory performance using the Morris Water Maze (MWM) test of Vorhees and Williams [26]. The swimming pool used for the test was 120 cm in diameter. The pool was filled to a height of 30 cm with water at 23°C (±1°C). The escape platform was fixed in a permanent position 2 cm under the water surface. The water tank was located in an experimental room and spatial reference cues (circle, rectangle, and triangle) around the pool remained fixed on the tank during the test. The pool was divided into four quadrants, namely zone NW (north-west), NE (north-east), SW (south-west) and SE (south-east). The quadrant housing the escape platform was defined as the target quadrant. A digital video camera (DCR-SR47; Sony Corporation, Tokyo, Japan) was installed two meters above the pool and was used to record the animals’ performance. The test was carried out in two phases as described below.

### Spatial acquisition

This trial evaluated the learning ability indicated by the decreasing escape latencies. All rats included in this study were selected from those who have passed the swimming ability test, had no physical defects or motor function deficits that could confound the study. The rats were trained in MWM for 5 consecutive days using a 4-trial-per-day regime. In each trial, the rats were placed in the pool facing the inner wall of the tank at one of four random starting points, such that the four positions were used each day. The rats were given 1 minute to locate the hidden platform, which is in the middle of the SW quadrant. Once the rat located the escape platform it was permitted to remain on it for 10 seconds. If the animals failed to find the platform, they were directed onto the platform. The escape latency was recorded by the video camera and analyzed by ANY-maze video tracking system software (Stoelting Co., USA).

### Probe trial

To estimate spatial memory retention, a probe trial was performed 1 day after the last acquisition day. In this phase, the platform was removed from the pool and rats were allowed to swim for 1 minute. The relative time spent by each rat in each quadrant was recorded and analyzed. The spatial accuracy of the animal is represented by the time the animal spent on looking for the platform in the target quadrant where the platform used to be during the acquisition phase.

### Fatty acid analysis

Total lipids from the experimental diets were extracted according to the methods described by [30] and modified by [31]. The experimental dets approximately 2 g were mixed with 40 ml of chloroform-methanol (2:1, v/v) containing butylated hydroxytoluene as antioxidant. Then, fatty acids methyl esters (FAME) were prepared using 0.66 N potassium hydroxide (KOH) in methanol and 14% methanolic boron trifluoride (BF_3_) (Sigma Chemical Co. St. Louis, Missouri, USA). The FAME were separated with an Agilent 6890A Series GC system (Agilent Technologies, Palo Alto, CA, USA) using a 30 m × 0.25 mm ID (0.20 μm film thickness) Supelco SP-2330 capillary column (Supelco, Inc., Bellefonte, PA, USA). The fatty acid proportions are expressed as percentage of total identified fatty acids. One microlitre of FAME was injected by an auto sampler into the chromatograph, equipped with a split/splitless injector and a flame ionization detector (FID) detector. The injector temperature was programmed at 250°C and the detector temperature was 300°C. The column temperature program initiated runs at 100°C, for 2 min, warmed to 170°C at 10°C /min, held for 2 min, warmed to 200°C at 7.5°C /min, and then held for 20 min to facilitate optimal separation. Identification of fatty acids was carried out by comparing relative FAME peak retention times of samples to standards obtained from Sigma (St. Louis, MO, USA).

### Gene expression

Following the behavioral test, all rats were anesthetized with ketamine-xylazine (80 mg/kg and 10 mg/kg intra peritoneal, respectively). The brain was quickly excised and the hippocampus immediately dissected, frozen in liquid nitrogen and stored at −80°C until RNA extraction. Total RNA was extracted from 100 mg of frozen tissue using the RNeasy®lipid tissue mini kit (Qiagen, Hilden, Germany) and DNase digestion was completed during RNA purification using the RNase-Free DNase set (Qiagen, Hilden, Germany) according to the manufacturer’s instructions. Total RNA integrity was checked by agarose gel electrophoresis and purity was determined by the 260/280 NM ratio of absorbance readings. Purified total RNA (1 μg) was reverse transcribed using a Quantitect® reverse transcription kit (Qiagen, Hilden, Germany) in accordance with the manufacturer’s recommended procedure. Real-time PCR was performed on a Rotor-Gene 3000 Real-Time PCR Detection System (Corbett Research, Sydney, Australia) using Quantifast® SYBR green PCR I kit (Qiagen, Hilden, Germany). The sequences of primers were as follows: β-actin, sense: 5^′^-CTGTGTTGTCCCTGTATGCC-3^′^ antisense: 5^′^-TAGATGGGCACAGTGTGGGT-3^′^; PPARα, sense: 5^′^-CGACAAGTGTGATCGAAGCTGCAAG-3^′^ antisense: 5^′^-GTTGAAGTTCTTCAGGTAGGCTTC-3^′^; PPARγ, sense: 5^′^-GCGGAGATCTCCAGTGATATC-3^′^ antisense: 5^′^-TCA GCGACTGGGACTTTTCT-3^′^. The β-actin was used as the reference gene to normalize the tested genes. The sequence specificity of each primer pair was confirmed using BLAST, and all primers were purchased through 1^st^ BASE oligonucleotide synthesis (Singapore). Each reactions (25 μl) were contained 12.5 μl SYBR green PCR mix, 1 μl diluted cDNA, 0.3 μM each of forward and reverse primers and RNase free water. Target genes were amplified through the following thermocycling program: 95°C for 10´, 40 PCR cycles at 95°C for 30˝, 60°C for 20˝ and 72°C for 20˝. Fluorescence was measured at every 15˝ to construct the melting curve. The specificity of the amplification product was further verified by electrophoresis on a 0.8% agarose gel and by DNA sequencing. A real-time PCR was conducted for each primer pair in which cDNA samples were substituted with dH_2_O to verify that exogenous DNA was not present. Additionally, 1 μg of RNA isolated by the procedure described above were substituted for cDNA in a real-time PCR reaction to confirm that there were no genomic DNA contaminants in the RNA samples. Both negative controls showed no amplification after 40 cycles. Efficiency of amplification was determined for each primer pair using serial dilutions. The relative expression of each gene, normalized to the reference gene, between treated and control samples were calculated using the relative expression software tool (REST) [32].

#### Statistical analysis

The data acquired for acquisition & probe trials in MWM was analyzed using a two-way repeated measure ANOVA. Maze performance parameters were compared across attempts, days, and consequently across treatment groups. Significantly different means were then elucidated using the Bonferroni test. All tests were conducted at 95% confidence level. Gene expression was calculated using the formula below and expressed as fold change between treatment and control groups.

(1) $$\text{Ratio}=\frac{{\left(E_{\text{target}}\right)}^{\Delta CT_{\text{target}}\left(\text{Control}-\text{Treatment}\right)}}{{\left(E_{\text{reference}}\right)}^{\Delta CT_{\mathrm{reference}}\left(\text{Control}-\text{Treatment}\right)}}$$

Gene expression data were checked for normality and homogeneity of variance using SPSS statistical software. Differences in mean values between the treatments and control were tested using one way ANOVA. Data were expressed as Means ± SE. Differences of *P* < 0.05 were considered to be significant.

## Competing interests

The authors declare that they have no competing interests.

## Authors’ contributions

All authors conceived the study, participated in the experiment design and drafted the manuscript.
